# Hybridization and the spread of the apple maggot fly, *Rhagoletis pomonella* (Diptera: Tephritidae), in the northwestern United States

**DOI:** 10.1111/eva.12298

**Published:** 2015-08-13

**Authors:** Tracy Arcella, Glen R Hood, Thomas H Q Powell, Sheina B Sim, Wee L Yee, Dietmar Schwarz, Scott P Egan, Robert B Goughnour, James J Smith, Jeffrey L Feder

**Affiliations:** 1 Department of Biological Sciences, University of Notre DameNotre Dame, IN, USA; 2 USDA-ARS, Yakima Agricultural Research LaboratoryWapato, WA, USA; 3 Department of Biology, Western Washington UniversityBellingham, WA, USA; 4 Advanced Diagnostics and Therapeutics, University of Notre DameNotre Dame, IN, USA; 5 Washington State University ExtensionVancouver, WA, USA; 6 Departments of Entomology & Lyman Briggs College, Michigan State UniversityE. Lansing, MI, USA; 7 Environmental Change Initiative, University of Notre DameNotre Dame, IN, USA

**Keywords:** introgression, insect pest, microsatellites, *Rhagoletis zephyria*, snowberries, Washington state

## Abstract

Hybridization may be an important process interjecting variation into insect populations enabling host plant shifts and the origin of new economic pests. Here, we examine whether hybridization between the native snowberry-infesting fruit fly *Rhagoletis zephyria* (Snow) and the introduced quarantine pest *R. pomonella* (Walsh) is occurring and may aid the spread of the latter into more arid commercial apple-growing regions of central Washington state, USA. Results for 19 microsatellites implied hybridization occurring at a rate of 1.44% per generation between the species. However, there was no evidence for increased hybridization in central Washington. Allele frequencies for seven microsatellites in *R. pomonella* were more ‘*R. zephyria*-like’ in central Washington, suggesting that genes conferring resistance to desiccation may be adaptively introgressing from *R. zephyria*. However, in only one case was the putatively introgressing allele from *R. zephyria* not found in *R. pomonella* in the eastern USA. Thus, many of the alleles changing in frequency may have been prestanding in the introduced *R. pomonella* population. The dynamics of hybridization are therefore complex and nuanced for *R. pomonella*, with various causes and factors, including introgression for a portion, but not all of the genome, potentially contributing to the pest insect's spread.

## Introduction

Hybridization between closely related species can provide insights into the nature of species boundaries and the speciation process (Barton and Hewitt 1985; Arnold 1992; Harrison 1993; Mallet 2005). Hybrid individuals can have lower fitness due to sterility, the breakup of locally adapted gene complexes (Dobzhansky and Pavlovsky 1958; Haddon 1984; Hubbard et al. 1992; Maeher and Caddick 1995; Levin et al. 1996), or a mismatch between hybrid phenotype and parental environments (Rundle 2002; Egan and Funk 2009). In other circumstances, hybrids may have higher fitness, which can lead to the creation of a hybrid swarm or a new, genetically distinct evolutionary lineage or species (Gallez and Gottlieb 1982; Rieseberg 1997; Seehausen 2004; Grant et al. 2005; Schwarz et al. 2005; Mavárez et al. 2006; Abbott et al. 2013). In other cases, hybrids themselves may not have higher fitness, but certain genes may be favored, resulting in the adaptive introgression of a subset of favorable alleles into the genetic background of the alternate parental population (Seehausen 2004; Mallet 2005).

Hybridization and introgression can also affect biological communities and ecosystems, particularly in light of global climate change where rapid adaptation to novel environmental conditions is prevalent (Scriber 2011, 2014; Pauls et al. 2013; Chown et al. 2014; Chunco 2014; Moran and Alexander 2014). The process may be particularly detrimental to ecosystems when one species involved is invasive. In this case, hybridization can contribute to the genetic extirpation of native species (Echelle and Connor 1989; Rhymer and Simberloff 1996; Huxel 1999) or help facilitate the spread of a modified form of the invader into previously unpopulated habitats (Ellstrand and Schierenbeck 2000; Perry et al. 2001; Arcella et al. 2013). In addition, if hybrids alter the local ecology or experience an escape from biotic factors normally constraining population densities, they can cause the loss of endemic biodiversity and ecosystem functions (Lodge et al. 2012).

One area where the detrimental consequences of hybridization and introgression may be underappreciated and understudied concerns the evolution of new pest insects (Diehl and Bush 1984; Kirk et al. 2013). In this instance, hybridization may increase levels of genetic and phenotypic variation to help enable the creation of new biotypes or races capable of shifting and differentially adapting to novel host plants of agricultural importance. Also, hybridization need not involve the evolution of novel host plant-related traits, *per se*, to create a new pest. Instead, it may facilitate adaptation to nonhost related biotic or abiotic conditions associated with the agricultural setting or climate change, allowing an insect to expand its ecology to become an economic threat.

**Figure 1:**
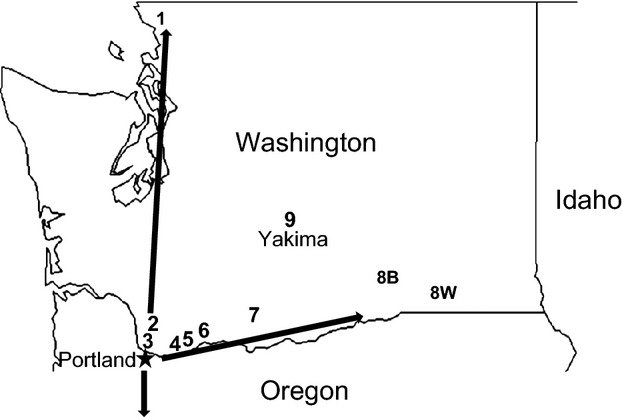
Map of the nine paired collection sites in Washington (WA) state genetically analyzed in the study. 1 = Bellingham; 2 = Vancouver, Washington State University campus; 3 = Vancouver; Burnt Bridge Creek Greenway; 4 = St. Cloud Park; 5 = Beacon Rock State Park; 6 = Home Valley; 7 = Klickitat; 8B = Burbank black hawthorn; 8W = Walla Walla snowberry; 9 = Tampico near Yakima. See Table S1 for site descriptions. Arrows denote spread of *R. pomonella* north and south along the western side of the Cascade Mountains and eastward into the Columbia River gorge following its putative introduction into Portland, OR. Black hawthorn-infesting populations of the fly have now encroached on the commercial apple-growing region of central WA centered in Yakima.

Here, we investigate the spread of the apple maggot fly, *Rhagoletis pomonella* Walsh, into the commercial apple-growing region of central Washington (WA) state, a $2.25 billion annual industry accounting for 75% of apple production in the United States (Mertz et al. 2013). Specifically, we examine whether hybridization of *R. pomonella* with its sibling species *R. zephyria* Snow is occurring and may be aiding the apple maggot in spreading into more arid and hotter central WA from mesic habitats west of the Cascade Mountains. The apple maggot fly was likely introduced to the Pacific Northwest (PNW) from its native range in the eastern United States (AliNiazee and Penrose 1981; AliNiazee and Westcott 1986; Brunner 1987; Tracewski et al. 1987; Hood et al. 2013; Sim 2013) where it is native to ancestral host hawthorn (*Crataegus* spp.). In the eastern United States, the fly shifted to introduced, domesticated apple *Malus domestica* ∼160 ya, forming a new host race, the initial step in ecological speciation with gene flow (Bush 1966, 1969; Feder et al. 1988, 1993; Egan et al. 2015). In the process, the apple-infesting race of *R. pomonella* became a major frugivorous pest of commercially grown apple. Female flies oviposit into ripening fruit growing in trees and, following egg hatch, larvae feed within fruit, causing damage and making the fruit unmarketable. More recently, *R. pomonella* has been detected in the PNW. It is believed the fly was originally introduced via larval-infested apples into the Portland, Oregon (OR) area (arrow 1 in Fig. 1), where the first report of apple infestation was made in 1979 (AliNiazee and Penrose 1981). Subsequently, *R. pomonella* spread north and south from Portland into WA and OR on the western side of the Cascade Mountains (arrow 2 in Fig. 1). The fly also moved eastward into the Columbia River Gorge and other passages in the Cascades (arrow 3 in Fig. 1) and has been encroaching on the commercial apple-growing region of central WA since the mid-1990s (Yee et al. 2012). Here, there is a zero infestation policy for apple export to foreign markets and for domestic consumption (WSDA 2001, Yee et al. 2012).

The spread of *R. pomonella* in the PNW is complicated by two factors of evolutionary and economic significance. First, *R. pomonella* in the PNW also attacks native black hawthorn, *C. douglasii* Lindley, and to a lesser degree native *C. suksdorffii* Sarg. and *C. douglasii* × *C. suksdorffii* hybrids, as well as the introduced ornamental hawthorn, *C. monogyna* Jacquin (Tracewski et al. 1987; Yee 2008; Yee and Goughnour 2008; Yee et al. 2012; Hood et al. 2013). The existence of black hawthorn-infesting populations of *R. pomonella* raises the possibility that the fly is native to the PNW. If this is true, then flies shifted from black hawthorn to apples and ornamental hawthorns when these latter two plants were introduced to the region. However, current knowledge of the geographic distribution of *R. pomonella* in the western United States is consistent with the introduction hypothesis. An extensive field survey of black hawthorns failed to detect *R. pomonella* in the PNW aside from areas where the fly was already known to occur (Hood et al. 2013). A more recent survey found *R. pomonella* infesting black hawthorn at an isolated site in Troy, Montana (Yee et al. 2015). However, flies were found infesting only one of 24 trees surveyed across a five-year period, a pattern consistent with a local introduction. Moreover, *R. pomonella* was first reported to attack *C. douglasii* in central WA in 2003 (Yee 2008; Yee et al. 2012). If the fly was native on black hawthorn and did not recently disperse into the area, then in all likelihood it should have been detected earlier.

Additionally, the pattern of genetic variation within *R. pomonella* conforms to the introduction hypothesis. Genetic diversity is reduced in the PNW compared to the eastern United States, but highest in and around the hypothesized area of introduction in Portland, OR (Sim 2013). Also, a genetic distance network based on microsatellites clustered all PNW populations of *R. pomonella* together and derived from a source in the midwestern United States where *C. monogyna* is absent. Finally, coalescence simulations of microsatellites estimate the age of the split between northwestern and eastern fly populations as only 13.6 years (Sim 2013), consistent with a recent historical timeframe for *R. pomonella* being introduced to the PNW.

Although *R. pomonella* is unlikely to be native on black hawthorn, the host currently appears to be the major conduit for spreading the fly into the apple-growing region of central WA (Yee et al. 2012). Moving eastward in the Columbia River Gorge and other mountain passes into central WA, environmental conditions become increasingly more arid and hotter. Feral apples are rare, and black hawthorn is the principal host for *R. pomonella* along creeks and streams, including those flowing into the Yakima River Valley, which is the center of the commercial apple industry (Yee et al. 2012). As yet, no exported apple from central WA has been found infested with a fly larva. However, computer simulations suggest that if unchecked, all apple-producing areas may be infested by *R. pomonella* in <30 years (Zhao et al. 2007).

The second factor complicating and potentially contributing to the spread of *R. pomonella* concerns *R. zephyria*, which infests snowberries (*Symphoricarpos* spp.). The two flies are parapatric in their distribution across the northern United States, overlapping extensively through the Midwest in Minnesota and Wisconsin (Bush 1966). In addition, *R. zephyria* is found in the northeastern United States into Canada, but exhibits a more patchy distribution across the region and may be non-native (Gavrilovic et al. 2007). The snowberry fly is also distributed through the northern plains states, where *R. pomonella* is not present, and westward into the PNW, where it is native and co-occurs with *R. pomonella*. In central WA, *R. zephyria* is problematic to commercial apple growers because it is abundant and difficult to definitively distinguish morphologically from the rarer *R. pomonella* when trapped in monitoring surveys (Westcott 1982; Yee et al. 2009, 2011, 2013). Due to the zero tolerance policy, misidentification of *R. zephyria* as *R. pomonella* is of concern because false positives can result in unnecessary quarantine measures being imposed at great cost to stakeholders, including growers and local and federal agencies (St. Jean et al. 2013).

Hybridization of *R. pomonella* with *R. zephyria* potentially poses a threat to the apple industry. Evidence suggests hybridization at a rate of 0.1% per generation between *R. pomonella* and *R. zephyria* in the eastern United States (Feder et al. 1999). In addition, *R. pomonella* and *R. zephyria* can be crossed to produce viable and fertile offspring in the laboratory, although at a reduced rate compared to pure parental matings (Yee and Goughnour 2011). Results from Green et al. (2013) suggest that a higher rate of hybridization is occurring in the PNW than eastern United States, with *R. zephyria* alleles extensively introgressing into *R. pomonella* in central WA. Here, low densities of *R. pomonella* infesting black hawthorn may encourage hybridization with the more abundant *R. zephyria*. Alleles common to *R. zephyria* elsewhere in WA were elevated in frequency in black hawthorn flies in the central apple-growing region of the state. However, the findings of Green et al. (2013) were based on a limited number of populations and individuals scored (60 flies combined from 2 locations in WA) and only 11 genetic markers. Nevertheless, the unidirectional pattern of introgression is consistent with transplant studies indicating that larval survivorship of *R. pomonella* is low in snowberry (Ragland et al. 2015). Hybridization could also help explain the difficulty in morphologically distinguishing *R. pomonella* from *R. zephyria* in central WA while contributing to the spread of a transgressive form (Rieseberg et al. 1999) of the apple maggot fly possessing features outside the normal phenotypic range of *R. pomonella* into the more arid and hotter apple-growing region of the state centered in Yakima.

Here, using a set of 19 microsatellite loci, we genotyped flies infesting black hawthorn and snowberry across nine pairs of sites where the flies co-occur or are in geographic proximity from west of the Cascade Mountains into central WA to test the hypotheses that: (i) *R. pomonella* and *R. zephyria* are hybridizing; and (ii) alleles from *R. zephyria* are introgressing into *R. pomonella* and potentially aiding its spread into the hotter and more arid central apple-growing region of the state. Comparisons of allele frequencies among these nine sites, as well as with potential source populations in the eastern United States, imply a complex pattern of low level hybridization and asymmetric introgression at some, but not all loci, implying a heterogeneous pattern of introgression throughout the genome.

## Materials and methods

### Sample collection

Infested fruit from snowberry bushes and black hawthorn trees were collected from nine pairs of ‘sympatric’ field sites in Washington state from July to September 2009 to 2012 where the flies co-occur less than 200 meters apart (Fig. 1; Table S1). The only exception was the ‘Burbank’ site where black hawthorn flies were collected in Burbank, WA, while the corresponding sample of snowberry flies was collected 50 km east near Walla Walla, WA. Fruits were transported back to the greenhouse of the Washington State University Extension Services, Clark County 78th street Heritage Farm, Vancouver, Washington, where they were then placed separately by host plant and site onto wire mesh racks held over plastic collecting tubs. Larvae were allowed to emerge from the fruit and pupate in the tubs. Pupae were collected on a daily basis and frozen immediately for later genetic analysis.

### Microsatellites

Flies were genotyped for 19 microsatellites originally developed for *R. pomonella* by Velez et al. (2006; see Table S2). The 19 microsatellites constitute a standard set of core loci analyzed for population differentiation in the *R. pomonella* sibling species group because they successfully PCR amplify and can be readily scored by multiplex genotyping for all taxa in the group (Michel et al. 2010; Cha et al. 2012; Powell et al. 2013, 2014). In addition, the 19 microsatellites are distributed across five of the six chromosomes (Michel et al. 2010) of the *Rhagoletis* genome (the small sixth dot chromosome is highly heterochromatic and currently does not contain a marker). Consequently, the genetic survey was not limited to one region of the genome but screened a representative portion for evidence of hybridization and introgression.

A total of 605 individuals collected from the nine sympatric sites (*n* = 278 from black hawthorn and *n* = 327 from snowberry) were genotyped in the study. Genomic DNA was isolated and purified from pupae or adults using PUREGENE extraction kits (Gentra Systems, Minneapolis, MN). PCR amplification and genotyping of microsatellites were carried out as previously described for *R. pomonella* in Michel et al. (2010) and Powell et al. (2013, 2014).

### Population genetic analyses

An unrooted neighbor-joining network was constructed based on overall Nei's (1972) genetic distances for the 19 microsatellites between populations using PowerMarker v3.25 (Liu and Muse 2005). For the Klickitat, WA population, two loci (p3 and p16) could not be scored due to a shortage of material and, thus, only 17 microsatellites were included in pairwise genetic distance measures calculated for this population. Bootstrap values were calculated based on 10 000 replicates across all loci.

To quantify genetic divergence between *R. pomonella* and *R. zephyria* and assess genotypes of individual flies for evidence of interhost migration and hybridization, we conducted a four-stage STRUCTURE analysis (v2.3.4; Pritchard et al. 2000). First, we tested for overall population structure for the entire microsatellite data set by conducting a blind (i.e., without *a priori* population information) STRUCTURE analysis for all 18 populations. For this analysis, we used the admixture and correlated alleles model, investigating *K* = 1–18 as possible numbers of genetically distinct subpopulations existing across sites. Three replicate runs of 250 000 MCMC generations, following a burn in period of 250 000 generations, were assessed for each *K* value.

Next, we conducted a more thorough second analysis of population subdivision for the 5 *K* values displaying the highest likelihood estimates in the initial STRUCTURE runs (*K* = 1–5). The follow-up analysis involved five replicate runs of 1 000 000 MCMC repetitions, following a burn in period of 500 000, for each *K* = 1–5 value. We used the *ΔK* method of Evanno et al. (2005) to identify the best value of *K* in the secondary STRUCTURE analysis.

Third, we assessed each of the nine sympatric *R. pomonella* and *R. zephyria* sites for evidence of population subdivision by conducting five blind runs for *K* = 1–2 (1 000 000 MCMC repetitions, following a burn in period of 500 000) separately for each paired site. Because the Evanno et al. (2005) method cannot be used for a comparison of *K* = 1 and *K* = 2, we used the mean Ln Likelihood estimates to evaluate the difference between these two values of *K*.

Fourth, potential migrants and hybrids were identified from STRUCTURE analyses performed at paired sympatric sites using the built-in function for population priors in cases when strong genetic structure exists between populations, as determined by step three above. The analyses for migrants and hybrids were conducted using a correlated allele frequency model and three replicate runs for *K* = 2 (1 000 000 MCMC repetitions, following a burn in period of 500 000) for each of three prior migration rates of 0.05 (the default), 0.01, and 0.1. The analysis produced posterior probabilities for each individual being derived from (i) cross of pure parental genotypes of natal host population origin (i.e., resident *R. pomonella* or *R. zephyria* flies infesting black hawthorn versus snowberry fruit, respectively); (ii) a pure parental cross of non-natal host origin (i.e., a fly whose mother was a migrant from the alternate host); (iii) a hybrid cross between *R. zephyria* and *R. pomonella* (F1 hybrid); and (iv) a cross of a hybrid individual with one or the other parental types (backcross). Migration rates (gene flow levels) per generation between hosts could then be estimated as the number of migrant genotypes plus half the number of F1 hybrids divided by the total number of flies scored. Hybridization rates per generation similarly could be estimated as the number of F1 hybrids divided by the total number of flies scored.

Mantel and partial Mantel tests for associations between Nei's genetic distances D between populations for microsatellites and their physical geographic distance and species identity (as a binary distance) were conducted using the package *vegan* (Oksanen et al. 2015) in R (R Development Core^2014^). Geographic distance matrices were produced using the R package *geosphere* (Hijmans 2014). Mantel tests were conducted based on 10 000 permutations and a Pearson correlation coefficient.

The overall mean and individual microsatellite locus allele frequency differences between *R. pomonella* and *R. zephyria* populations at the nine paired sites were also analyzed by linear regression against their geographic distance to the Tampico site near Yakima in central WA in R (R Development Core). To test the hypothesis that the genetic difference between species should decrease with proximity to Yakima given adaptive introgression from *R. zephyria* into *R. pomonella*, nonparametric, Monte Carlo simulations were used to determine significance levels for the regressions. In contrast to the Mantel tests which considered geographic distance between populations *per se* as the determinant of genetic differentiation regardless of ecology (i.e., isolation by distance), the linear regression with distance to Yakima tested for microsatellite convergence of *R. pomonella* with *R. zephyria* along the primary axes of climatic change (drier and hotter environmental conditions) varying from western to central WA hypothesized to underlie the adaptive introgression of alleles into *R. pomonella*. To test for statistical significance, microsatellite genotypes were randomly assigned within each species among the nine sites to determine how often a positive regression coefficient as large as that observed between allele frequency differences between the species at sites and the distance of sites to Yakima could be generated by chance. In addition, linear regressions were performed to test for an association between mean allele frequency differences and three environmental factors: (i) mean precipitation at sites from July to October, (ii) average high temperature at sites in July, and (iii) average low temperature at sites in January, as measured at recording stations near each site from 1981 to 2010, as listed on the Web sites for US Climate Data ( http://www.usclimatedata.com) and the Western Regional Climate Center ( http://www.wrcc.dri.edu).

## Results

### Species differences

None of the 19 microsatellites scored displayed a diagnostic difference between *R. pomonella* and *R. zephyria* (i.e., no one allele or set of alleles was fixed at a frequency of 1.0 in one of the species and absent in the other; Table S3). Nevertheless, several microsatellites displayed large frequency differences between the species. For example, allele 226 at locus p7 was present at a frequency of at least 0.50 in all *R. zephyria* populations (range 0.50–0.96), while its highest frequency in any of the nine *R. pomonella* populations surveyed was 0.19. Conversely, allele 150 at locus p27 was present at a frequency of at least 0.18 in all *R. pomonella* populations, while its highest frequency in any *R. zephyria* population was 0.03. There were also a total of 196 private alleles for the 19 microsatellites found in one or more population of one of the species and not the other (Table S3). These private alleles were generally not observed at high frequency within populations (<0.10) and were usually not found across all nine populations of a single species. Indeed, almost half of the private alleles were present in only one of the 18 host-associated populations surveyed in WA. *Rhagoletis pomonella* possessed a higher percentage of private alleles (129 of 196 total = 65.8%) than *R. zephyria* (*X*^2^ = 19.6, *P* = 0.00001, df = 1), consistent with the hypothesis of gene flow being primarily in the direction of snowberry into apple maggot fly populations. However, the difference could also be explained by larger effective population sizes and/or higher microsatellite mutation rates in *R. pomonella,* but given the history of recent introduction and generally lower densities of the apple maggot in the PNW, these two hypotheses seem less likely. Moreover, we genotyped fewer black hawthorn (*n* = 278) than snowberry-infesting (*n* = 327) flies, biasing the detection of private alleles in the direction of *R. zephyria*.

**Figure 2:**
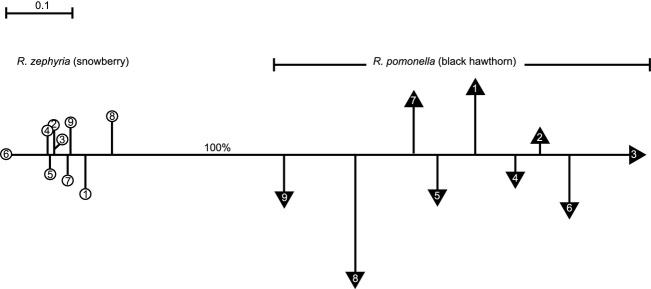
Neighbor-joining network for the nine paired black hawthorn-infesting *R. pomonella* (unfilled circles) and snowberry-infesting *R. zephyria* populations (dark triangles) in WA based on Nei's overall genetic distances for 19 microsatellite loci. See Fig. 1 legend and Table S1 for descriptions of the nine paired sites designated. Bootstrap support values based on 10 000 replicates are given for the node separating *R. pomonella* and *R. zephyria* populations.

**Figure 3:**
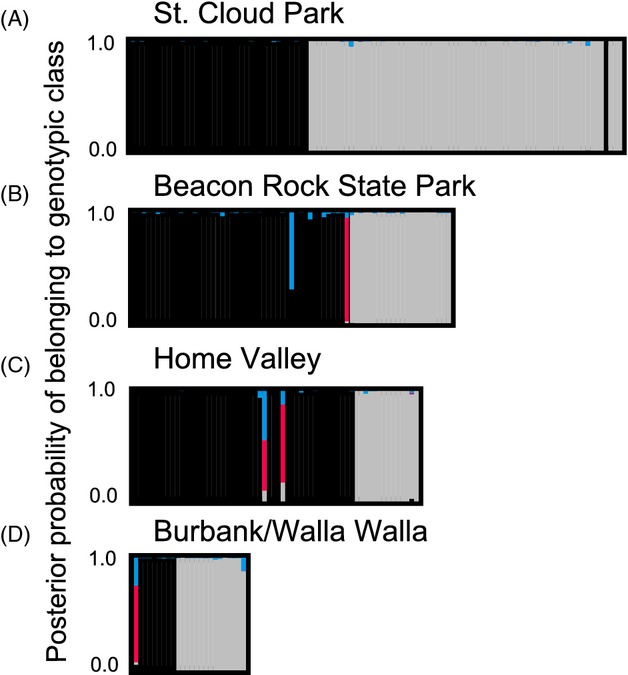
STRUCTURE bar plots for four paired sites at (A) St. Cloud Park; (B) Beacon Rock State Park; (C) Home Valley; and (D) Burbank/Walla Walla, WA, depicting posterior probabilities of individual *R. pomonella* black hawthorn fly genotypes (on left) and *R. zephyria* snowberry fly genotypes (on right) belonging to one of four genotypic classes: pure *R. pomonella* origin (black), pure *R. zephyria* (light gray), F1 hybrid (red), or backcross (blue), based on genotypes at 19 microsatellite loci. Bars along the *x*-axis represent individual flies.

Despite the lack of a fixed difference, microsatellite allele frequencies differed sufficiently enough such that all nine *R. zephyria* and nine *R. pomonella* populations clustered distinctly and separately from one another with 100% bootstrap support in the neighbor-joining (NJ) network (Fig. 2). In addition, a blind STRUCTURE analysis conducted by combining flies from all nine paired sympatric sites gave a best fit of *K* = 2 across the PNW, corresponding to the species *R. zephyria* and *R. pomonella* (Table S4; Fig. S1 and S2). Similarly, a model of *K* = 2 was supported for each of the nine paired sites considered separately, representing the two taxa (see Fig. 3A–D for STRUCTURE plots for the St. Cloud Park, Beacon Rock State Park, Home Valley, and Burbank/Walla Walla, and Fig. S2 for STRUCTURE plots for all sites, and Table S5 for mean Ln Likelihoods).

### Inbreeding coefficients within populations

Microsatellite genotypes within populations of both species tended to be slightly heterozygote deficient, with low positive mean inbreeding coefficients (*f*) across loci (Table S6). There appeared to be considerable variation in *f* values among loci. However, only one population, *R. pomonella* from Devine (site #3), had a consistently positive inbreeding coefficient such that the standard deviation of *f* values across loci did not overlap zero. *Rhagoletis zephyria* and *R. pomonella* did not differ in *f* (paired *t*-test; *t* = −0.331; *P* = 0.7491; df = 8), but *R. zephyria* populations did have lower observed heterozygosity than *R. pomonella* (Table S3; paired *t*-test; *t* = 6.68; *P* = 0.00016; df = 8), consistent with the generally lower level of polymorphism found in the former species (see above). The results imply that deviations from random mating were not significant within local populations of either *R. zephyria* or *R. pomonella* and that the 19 microsatellites scored in the study did not possess high frequencies of null alleles.

### Hybridization and gene flow

The finding of substantial population structure between black hawthorn versus snowberry-infesting fly populations at each of the nine paired sympatric sites allowed for posterior probabilities to be estimated for individuals to assess their genetic ancestry. Based on these probabilities, we found evidence for ongoing migration, gene flow, hybridization, and introgression between *R. pomonella* and *R. zephyria* in WA (Figs 3 and S2). The results were qualitatively similar for the three different priors used for the migration rate (*m* = 0.05, 0.01, and 0.1), with the exception that more backcross individuals were identified for the *m* = 0.1 model. We therefore present the finding generated from the most conservative *m* = 0.01 model. One of the 46 flies reared from black hawthorn at the Beacon Rock (Fig. 3B), two of the 48 black hawthorn-origin flies from the Home Valley (Fig. 3C), and one of the nine black hawthorn flies from the Burbank/Walla Walla (Fig. 3D) possessed multilocus microsatellite genotypes making them most likely to be F1 hybrids. One black hawthorn-origin fly from Beacon Rock had a genotype with a high posterior probability of being a later generation F2 or backcross hybrid (Fig. 3B). In addition, one of 71 individual reared from snowberry at the St. Cloud had a posterior probability for being a parental *R. pomonella* migrant (Fig. 3A). None of the identified migrants or hybrids had greater than two microsatellite loci with missing data. Based on these data, we estimated the hybridization rate of *R. pomonella* and *R. zephyria* on black hawthorn as 1.44% per generation (= 4 F1 hybrids/278 black hawthorn-origin flies genotyped in the study). In contrast, no evidence for hybridization was detected between the two species on snowberries. The estimated rate of migration and gene flow from the snowberry into black hawthorn-infesting fly populations was 0.0072 per generation, while it was 0.0031 in the reverse direction.

### Isolation by distance

In a partial Mantel across all populations after species identity was accounted for, geographic distance was not significantly related to population genetic distance (Mantel *r* = 0.032; *P* = 0.2785). Geographic distance was correlated with population genetic distance for *R. zephyria* populations analyzed separately (Mantel *r* = 0.7457; *P* = 0.0067), but not for *R. pomonella* populations (Mantel *r* = 0.4924; *P* = 0.11689).

### Genetic divergence in relation to Yakima

**Figure 4:**
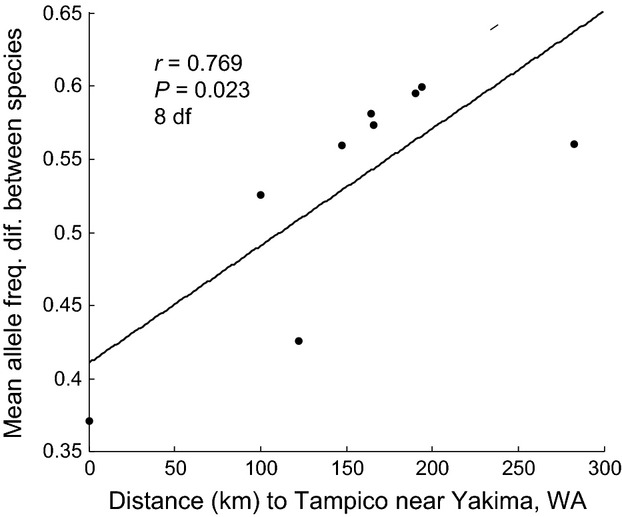
Association between mean allele frequency difference for 19 microsatellite loci between *R. zephyria* and *R. pomonella* populations at each of the nine paired sites plotted against each pairs geographic distance to the Tampico unincorporated community near Yakima, WA. Best fit line added to illustrate association. Note: the mean allele frequency difference at the Klickitat site was based on only 17 loci.

Microsatellite allele frequency differences between *R. pomonella* and *R. zephyria* populations across the nine paired sites were significantly related to geographic distance to Tampico near Yakima in central WA (Table 1, Fig. 4). Seven loci designated p4, p7, p16, p18, p25, p46, and p80, as well as the overall pattern for all 19 loci scored in the study, displayed significant trends for black hawthorn flies to become more ‘snowberry-like’ in their frequencies with geographic proximity to Yakima (Table 1, Fig. 4). As mean July to October precipitation also decreases with proximity to Yakima, overall microsatellite genetic distance for black hawthorn flies was also significantly correlated with rainfall (*r* = 0.729, *P* = 0.026, df = 8), consistent with the desiccation hypothesis. Mean allele frequency differences were also strongly correlated with summer high temperatures (*r* = −0.752, *P* = 0.019, df = 8) and winter low temperatures (*r* = 0.958, *P* < 0.0001, df = 8). The seven significant loci displaying a relationship with distance to Yakima were distributed across all five of the chromosomes for which genetic markers were scored (Table 1), implying that the pattern was not restricted to just one particular region of the genome. The increased genetic similarity of *R. pomonella* to *R. zephyria* was also reflected in the Burbank and Yakima black hawthorn populations (sites 8 and 9) being closest to snowberry flies in the genetic distance network (Fig. 2). However, allele frequencies for the remaining 12 microsatellites did not differ significantly with geographic distance, with two loci (p66 and p70) having negative, but not significant, regression coefficients (Table 1). Also, the seven significant loci described above also differed significantly between *R. pomonella* and *R. zephyria* Burbank and Yakima in central WA (Table S3). Moreover, with the exception of locus p16, every microsatellite possessed at least one private allele present in *R. zephyria*, but not in any of the nine co-occurring *R. pomonella* populations scored, even for those loci displaying significant frequency convergence between the two species with geographic proximity to Yakima. In addition, there were a total of 41 instances in which an allele was present at a microsatellite locus in only two of the total of 18 host-associated populations surveyed in the study. If introgression was appreciable between local *R. pomonella* and *R. zephyria* populations, then we would expect to observe several instances in which the rare allele was shared between the taxa at sympatric sites and not present anywhere else. However, in only one of the 41 cases was this so, which was lower than the null expectation of 1.2 (= [9/306] × 41). Consequently, while the microsatellites implied hybridization, gene flow, and a degree of genetic converge between *R. zephyria* and *R. pomonella* approaching central WA, there was also evidence for introgression being restricted between the species despite hybridization.

**Table 1:** Regression coefficients (*r*) for microsatellite loci of allele frequency differences between co-occurring *R. zephyria* and *R. pomonella* populations at nine paired sites against geographic distance of each location to the Yakima County site at Rosyln, WA. Also given in the column designated ‘Chr #’ are the chromosome (linkage group) assignments that each microsatellite locus has been mapped to in the *Rhagoletis* genome (Michel et al. 2010) and significance levels (*P* values) for regressions (significant loci are bolded). Significance was determined by nonparametric Mote Carlo simulations as described in the Materials and methods

Locus	Chr #	*r*	*P*
p71	1	0.549	0.085
p37	1	0.169	0.355
**p4**	**1**	**0.796**	**0.020**
p3	1	0.607	0.107
p70	2	−0.115	0.574
**p46**	**2**	**0.707**	**0.034**
p73	2	0.097	0.409
**p7**	**3**	**0.655**	**0.049**
**p80**	**3**	**0.671**	**0.048**
**p16**	**3**	**0.766**	**0.045**
p66	3	−0.507	0.864
p11	4	0.149	0.376
p29	4	0.483	0.140
**p25**	**4**	**0.772**	**0.017**
p50	4	0.200	0.339
p60	4	0.508	0.141
**p18**	**5**	**0.847**	**0.007**
p9	5	0.001	0.502
p27	5	0.303	0.276
**All loci**		**0.769**	**0.023**

## Discussion

Our results imply a more nuanced scenario than envisioned by Green et al. (2013) for the potential role that hybridization may be playing in the spread of *R. pomonella* into the more arid apple-growing region of central Washington. We found no evidence for a hybrid swarm between *R. pomonella* and *R. zephyria* in central WA. However, the STRUCTURE analysis implied mainly unidirectional hybridization between *R. zephyria* into *R. pomonella* on black hawthorn occurring at a rate of 1.44% per generation (4 F1 hybrids of a total of 278 black hawthorn-infesting flies scored), which is higher than that of 0.1% inferred from previous work (Feder et al. 1999). Nevertheless, there was no indication for a dramatically increased rate of hybridization in central WA compared to elsewhere. One of the likely F1 hybrids was detected at the Burbank site in central Washington, but none were evident at the Yakima site in the heart of apple production region of the state (Fig. S1). Moreover, one putative F1 hybrid was found at the Beacon Rock site and two putative hybrids were present at the Home Valley site in the Columbia River Gorge. Thus, while hybridization appears to be occurring and may disproportionately result in an influx of *R. zephyria* alleles into *R. pomonella*, the microsatellites imply that the rate is not greatly elevated in central WA.

There was a general tendency for *R. pomonella* to become genetically more similar to, but still distinct from, *R. zephyria*, approaching Yakima in central WA. There was no overall pattern of isolation by distance for *R. pomonella*, however. Thus, geographic distance *per se* between black hawthorn sites was not significantly related to the degree to which they were genetically diverged. Rather, it was the distance of sites from Yakima along the axis of varying environmental conditions that differentiated fly populations. The results therefore suggest that alleles associated with the snowberry fly may be favored in black hawthorn populations in the central WA region, possibly due to selection for increase desiccation resistance, but not in black hawthorn flies in western WA. However, this trend was not apparent for every locus. Indeed, many microsatellites suggest rather restricted hybridization and little gene flow locally between the two species. It may therefore be that much of the genome is not freely introgressing between *R. pomonella* and *R. zephyria*. Thus, although hybridization between the two species in the PNW may generally be higher than previously appreciated (1.44%), the effective introgression rate may be low, while potentially contributing to the adaptive process for *R. pomonella*. Such a scenario seems more in line with studies showing that larval survivorship is not high in reciprocal host transplant experiments (Ragland et al. 2015) and that a degree of postzygotic isolation also exists between *R. pomonella* and *R. zephyria* (Yee and Goughnour 2011). Future studies investigating the feeding performance the genetic patterns of surviving individuals from reciprocally transplanted F1 *R. pomonella* × *R. zephyria* hybrids and backcrossed individuals would be of particular interest.

Comparisons of microsatellite variation between *R. pomonella* and *R. zephyria* in the PNW to that for *R. pomonella* in its native range in the eastern United States also implies more subtle dynamics of introgression between the two species. Combining microsatellite data from Michel et al. (2010) and Powell et al. (2013, 2014) with those from the current study, we identified a total of 50 alleles in western *R. zephyria* not found in *R. pomonella* populations in the midwestern United States, the area from where the apple maggot fly was likely introduced (Sim 2013). These *R. zephyria* alleles ‘private’ to the northwest are insightful because they provide a means to gauge the degree and effectiveness of gene flow from *R. zephyria* into *R. pomonella* in the region. If these *R. zephyria* alleles are also present in western *R. pomonella* populations but not in the Midwest, then it is likely they introgressed from *R. zephyria*. It would seem improbable that if a high proportion of *R. zephyria* variants are present in western but not eastern *R. pomonella*, that they could all have been independently derived in western apple maggot flies following their recent introduction, but rather represent homoplasy, especially for alleles found at high frequencies in black hawthorn-infesting fly populations. Nor could the pattern be due to founder effects associated with the introduction of *R. pomonella* to the western United States because the alleles are not present in the midwestern United States.

In total, we detected 19 of 50 ‘unique *R. zephyria*’ alleles (38%) in black hawthorn populations in WA, implying introgression. However, most of these shared alleles between the two species in WA were present at low frequencies (<0.05%) and in only a subset of the nine *R. pomonella* populations surveyed. Indeed, in only eight of the 19 instances was the putatively introgressed *R. zephyria* allele found at modest to high frequencies in a majority of the *R. pomonella* populations sampled (allele 143 at locus p3, alleles 301 and 315 at locus p18, allele 156 at locus p50, alleles 212 and 232 at locus p66, and alleles 207 and 211 at locus p80). Moreover, of these eight variants, only allele 301 at microsatellite p18 showed a clear pattern of increased frequency in central WA, although this was the locus displaying the most pronounced convergence with *R. zephyria* in the study. For the other significant microsatellites positively varying with proximity to Yakima, patterns were diverse. First, no shared and presumably introgressed unique *R. zephyria* allele was present at four loci (p4, p7, p25, and p46); second, for one locus (p16), a shared allele (292) was present at low frequency (0.0132) and only in one *R. pomonella* population (WSU); third, for the remaining locus (p80), there were several shared alleles distributed widely across black hawthorn fly populations, but not obviously increasing in frequency in central WA. Thus, for the majority of loci showing convergence with *R. zephyria* in central WA, the alleles can also be found in *R. pomonella* populations in the midwestern United States. As such, these genes may represent standing variation in *R. pomonella* that was present at the time of the fly's introduction prior to its spread into central WA. The origin of these alleles in *R. pomonella* could conceivably trace to past historical gene flow between *R. zephyria* and *R. pomonella* predating the western introduction. However, there is currently no evidence from the microsatellites that these genes recently introgressed into *R. pomonella* to facilitate the fly's movement into central WA. Proof or refutation of recent introgression will require much more detailed DNA sequence analysis. Consequently, certain variants at certain microsatellites appear to have introgressed and may be adaptive in *R. pomonella* (in particular, allele 301 at microsatellite p18), while other instances of observed geographic variation in WA could involve standing genetic variation.

The story of hybridization and its role in the adaptive spread of the apple maggot fly in WA may therefore be more complicated than one involving widespread introgression of *R. zephyria* alleles into introduced *R. pomonella* populations. In central WA, larvae finish feeding, exit fruit, burrow into the soil, and form puparia during the hottest and driest period of the year. Studies suggest that *R. zephyria* pupae have a greater tolerance to desiccation than *R. pomonella* (Neilson 1964; Tracewski and Brunner 1987). Consistent with the desiccation hypothesis, we found an overall trend for *R. pomonella* populations to become more ‘*R. zephyria*-like’ in their allele frequencies with decreased rainfall in proximity to Yakima. It is therefore possible that selection for desiccation resistance alleles introgressing from *R. zephyria* are actively adapting *R. pomonella* to the harsher environmental conditions for pupae in central WA, facilitating its spread into the commercial apple regions of the state. In addition, genetic similarity between *R. pomonella* and *R. zephyria* also increases with increasing high temperature at sites in July and with lower mean temperature in January, suggesting that diapause life history timing or another phenotype related to temperature rather than desiccation *per se* could alternatively be the source of selection. It remains to be determined, however, whether the observed relationships are causative with respect to desiccation resistance or merely represent correlations with aridity. Resolving these issues will require coupling selection experiments in *R. pomonella* on desiccation resistance and diapause traits to connect allelic variants with survivorship differences to these evolutionary histories. Such studies are also necessary to discount neutral isolation by distance due to variable gene flow and drift among populations generating the observed pattern of introgression. In this regard, there is a general decrease of genetic diversity in *R. pomonella* populations from the putative site of introduction in Portland, OR eastward that could reflect decreasing population sizes associated with the fly's spread toward central WA (Sim 2013). However, allele frequencies of the seven microsatellites showing a significant relationship with proximity to Yakima in central WA (Table 1; Fig. 4) were all in the direction of being more ‘*R. zephyria*-like’ compared to populations in the western portion of the state, implying a deterministic rather than demographic explanation for the observed patterns of introgression.

Strong population genetic differentiation is maintained between *R. pomonella* and *R. zephyria*, despite a gross hybridization rate (∼1.44%) that should lead to the eventual erosion of differentiation at equilibrium. The data presented here therefore add to the evidence that many ‘good’ species persist in the face of considerable hybridization (Mallet 2005) and that population genetic differentiation is not always a straightforward function of the migration rate. Factors including positive and negative selection acting differentially on genes, and structural features of the genome, such as inversion polymorphism in *R. pomonella* (Feder et al. 2003a) and the general lack of recombination in Dipteran males, can all disassociate rates of hybridization and effective migration (introgression) variably across the genome between populations.

In conclusion, there are a growing number of examples documenting differential adaptation of insect populations to commercially grown host plants (Kirk et al. 2013). Standing genetic variation is often cited as one important factor facilitating insect shifts to novel host species (Barrett and Schluter 2008; Kirk et al. 2013). Hybridization could therefore be an important and understudied process interjecting genetic variation into insect populations enabling host shifts and the genesis of new economic pests. Adaptive introgression could be of particular concern in an era of rapid global climate change and increasing species introductions, as populations encounter novel environmental conditions on an accelerated basis (Balint et al. 2011; Scriber 2011, 2014; Pauls et al. 2013; Chown et al. 2014; Chunco 2014; Moran and Alexander 2014). Previous studies of hawthorn-infesting populations of *R. pomonella* have suggested that past cycles of geographic isolation, genetic differentiation, secondary contact, and subsequent hybridization have contributed to the adaptive radiation of members of the *R. pomonella* sibling species complex onto a variety of novel host plants (Feder et al. 2003b, 2005; Xie et al. 2008). In addition, hybridization between *R. zephyria* and *R. mendax* may have given rise to a new honeysuckle-infesting population of *Rhagoletis* attacking *Lonicera* (Schwarz et al. 2005). Here, we investigated whether ongoing hybridization between *R. zephyria* and the recently introduced *R. pomonella* is contributing to the spread and adaptation of the apple maggot fly to harsher environmental conditions found in the commercial apple-growing region of central WA. Our results imply that standing variation may be important for *R. pomonella*'s establishment in central WA. We found evidence that some of this variation could have its roots in *R. zephyria* and have recently introgressed into western *R. pomonella* populations, while a significant portion may have a deeper history in *R. pomonella* predating its introduction. Thus, a diversity of causes and factors, including hybridization, likely underlie the story of *R. pomonella*'s invasion and spread in the western United States.

## Data archiving statement

Data for this study are included in Table S3.

## Supporting Information

**Figure S1.** STRUCTURE bar plot for analysis of all 18 populations for *K* = 2, the most well-supported number of diverged, non-randomly mating demes based on 19 microsatellites.

**Figure S2.** STRUCTURE bar plots for nine paired sites at (A) Bellingham; (B) WSU; (C) Devine; (D) St. Cloud Park; (E) Beacon Rock State Park; (F) Home Valley; (G) Klickitat; (H) Burbank/Walla Walla, WA; (I) Yakima; depicting posterior probabilities of individual *R. pomonella* black hawthorn fly genotypes (on left) and *R. zephyria* snowberry fly genotypes (on right) belonging to one of four genotypic classes: pure *R. pomonella* origin (black), pure *R. zephyria* (light grey), F1 hybrid (red), or backcross (blue), based on genotypes at 19 microsatellite loci. Bars along the *x*-axis represent individual flies.

**Table S1.** Location of collecting sites in Washington state, U.S.A., for which flies were genetically analyzed in the study.

**Table S2.** List of microsatellites analyzed, their Genbank accession numbers, the chromosomal location of each locus in the *Rhagoletis* genome (Michel et al. 2010), and the primer pairs used to PCR amplify the microsatellites.

**Table S3.** Allele frequencies for 19 microsatellite loci scored in nine paired populations of Rhagoletis zephyria flies colected from during the 2009–2011 field seasons.

**Table S4.** Mean estimated Ln likelihood, standard deviation, and ΔK (Evanno et al. 2005) calculated across five replicates of STRUCTURE analysis for all eighteen populations for *K* = 1–5, using a burn-in of 500 000 followed by 1 000 000 MCMC repetitions under a correlated allele frequencies with admixture model.

**Table S5.** Mean estimated Ln likelihood and standard deviation across five replicates of a STRUCTURE analysis of paired local *R. pomonella* and *R. zephyria* populations for *K* = 1 and *K* = 2, using a burn-in of 500 000 followed by 1 000 000 MCMC repetitions under a correlated allele frequency with admixture model.

**Table S6.** Mean demic inbreeding coefficient (*f*) and stand deviation (σ) across all 19 loci for each of 9 paired *R. pomonella* and *R. zephyria* population.
