# Supplementary figures and images for: Hybridization and the spread of the apple maggot fly, Rhagoletis pomonella (Diptera: Tephritidae), in the northwestern United States
Source: Evol Appl. 2015 Aug 13;8(8):834–46. doi: 10.1111/eva.12298 (PMC4561572; doi:10.1111/eva.12298)

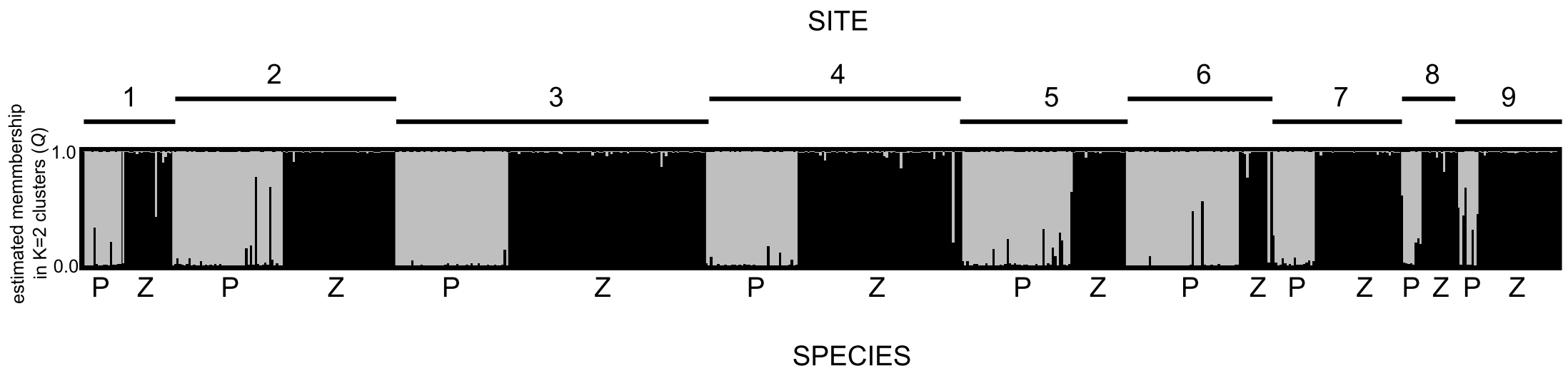

Supplement: Supplementary file 1 — Figure S1. STRUCTURE bar plot for analysis of all 18 populations for K = 2, the most well-supported number of diverged, non-randomly mating demes based on 19 microsatellites. [file eva0008-0834-sd1.pdf]

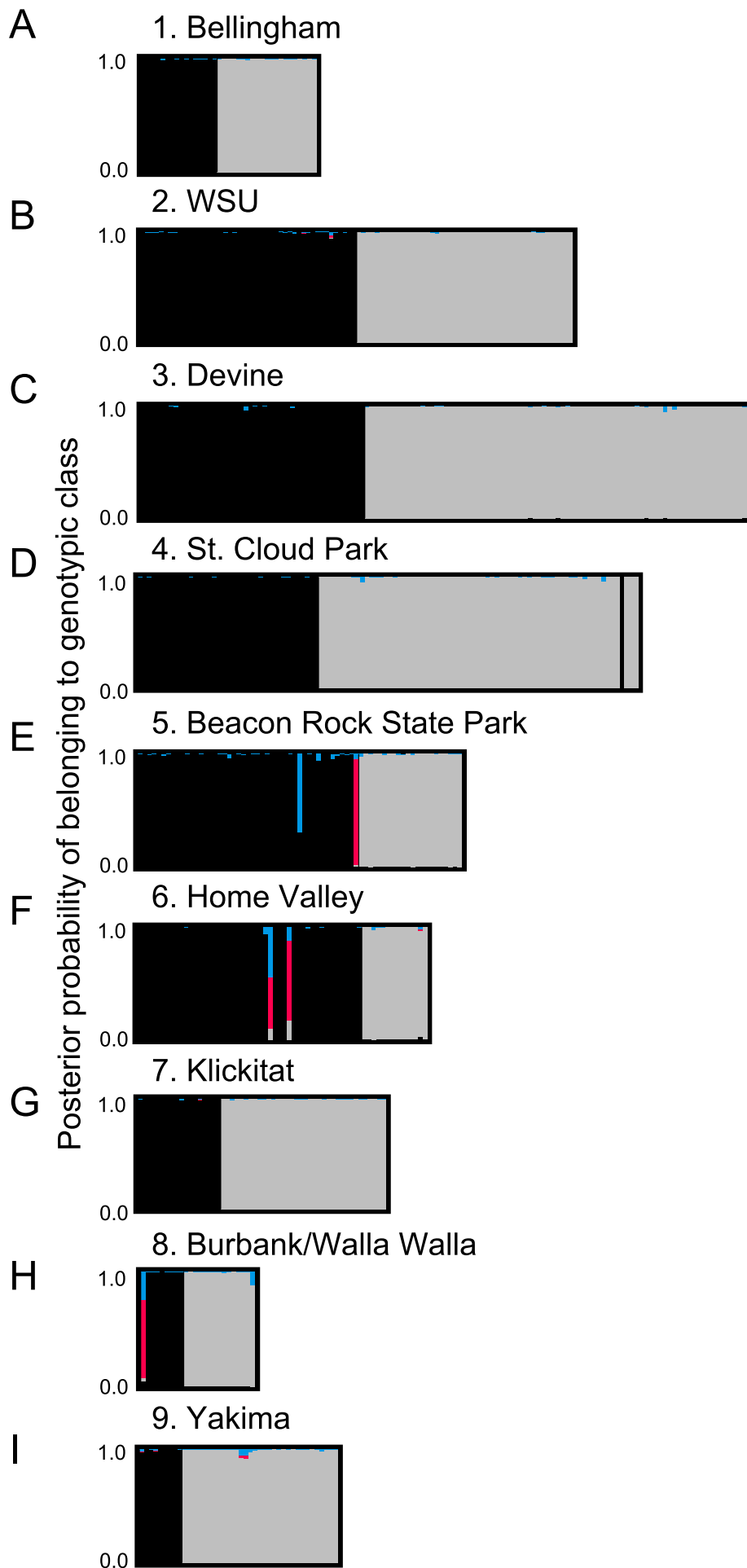

Supplement: Supplementary file 2 — Figure S2. STRUCTURE bar plots for nine paired sites at (A) Bellingham; (B) WSU; (C) Devine; (D) St. Cloud Park; (E) Beacon Rock State Park; (F) Home Valley; (G) Klickitat; (H) Burbank/Walla Walla, WA; (I) Yakima; depicting posterior probabilities of individual R. pomonella black hawthorn fly genotypes (on left) and R. zephyria snowberry fly genotypes (on right) belonging to one of four genotypic classes: pure R. pomonella origin (black), pure R. zephyria (light grey), F1 hybrid (red), or backcross (blue), based on genotypes at 19 microsatellite loci. Bars along the x-axis represent individual flies. [file eva0008-0834-sd2.pdf]
