# Supplementary material for: Hybridization and the spread of the apple maggot fly, Rhagoletis pomonella (Diptera: Tephritidae), in the northwestern United States
Source: Evol Appl. 2015 Aug 13;8(8):834–46. doi: 10.1111/eva.12298 (PMC4561572; doi:10.1111/eva.12298)
Supplement: Supplementary file 3 — Table S1. Location of collecting sites in Washington state, U.S.A., for which flies were genetically analyzed in the study. [file eva0008-0834-sd3.docx]

**Support Information Table S1.** Location of collecting sites in Washington state, U.S.A., for which flies were genetically analyzed in the study.

| Site # | Site | Lat. Long. | | |  |  |
| --- | --- | --- | --- | --- | --- | --- |
| 1. Bellingham | | | 48.74 | -122.48 | | |
| 2. Vancouver, Washington State Univ. (WSU) | | | 45.73 | -122.63 | |  |
| 3. Vancouver, Burnt Bridge Creek Greenway, Devine St. | | | 45.63 | -122.60 | |  |
| 4. Skamania, St Cloud Park | | | 45.60 | -122.11 | |  |
| 5. Beacon Rock State Park | | | 45.65 | -122.01 | |  |
| 6. Home Valley | | | 45.71 | -121.78 | |  |
| 7. Klickitat | | | 45.89 | -120.71 | |  |
| 8. Burbank (*R. pomonella* population)  Walla Walla (*R. zephyria* population) | | | 46.20  46.03 | -119.01  -118.20 | |  |
| 9. Tampico unincorporated community near Yakima | | | 46.61 | -120.50 | |  |
