# Supplementary material for: Hybridization and the spread of the apple maggot fly, Rhagoletis pomonella (Diptera: Tephritidae), in the northwestern United States
Source: Evol Appl. 2015 Aug 13;8(8):834–46. doi: 10.1111/eva.12298 (PMC4561572; doi:10.1111/eva.12298)
Supplement: Supplementary file 4 — Table S2. List of microsatellites analyzed, their Genbank accession numbers, the chromosomal location of each locus in the Rhagoletis genome (Michel et al. 2010), and the primer pairs used to PCR amplify the microsatellites. [file eva0008-0834-sd4.docx]

**Supporting Information Table S2**. List of microsatellites analyzed, their Genbank accession numbers, the chromosomal location of each locus in the *Rhagoletis* genome (Michel et al. 2010), and the primer pairs used to PCR amplify the microsatellites.

| Locus designation | Genbank accession number | Chromosome # | Primer sequences (5' to 3') |
| --- | --- | --- | --- |
| p03 | AY734887 | 1 | TCCACTCAAATACGGCAACA |
|  |  |  | GCAGCCGATCTTTTCGTCTA |
| p04 | AY734888 | 1 | GCAAGCGAGTCGTAATCACA |
|  |  |  | CCCTCATCATTGTGGTCCTC |
| p07 | AY734891 | 3 | CATTGGCAACGCTAGTTCAA |
|  |  |  | GCGCTGAAACCATGAAAAAT |
| p09 | AY734893 | 5 | CGGCAGGTAAATGACCAAAA |
|  |  |  | GCAATGACCGTTGGCTATTA |
| p11 | AY734895 | 4 | ATGCAGCCATGACTGAGATG |
|  |  |  | TGGAAAGTAATTTCACAAAGGCTA |
| p16 | AY734900 | 3 | CGCTTTAGATTTTCGCTACACA |
|  |  |  | ACGCAGTGCCAAATCTTCTT |
| p18 | AY734902 | 5 | CCCAATGTCCCGTAAACTTC |
|  |  |  | TTCACTCAATGCCCATTTCA |
| p25 | AY734909 | 4 | ATGACATTCGCTACGGGGTA |
|  |  |  | TCTCGGAGAGTGGCAGTTTT |
| p27 | AY734911 | 5 | TTCTCACATTTTCGCGTTTG |
|  |  |  | CTGGCCAATGCATAAATCCT |
| p29 | AY734913 | 4 | TCCATGTGTGCCAGAACATT |
|  |  |  | GACGTTATTTCGCTCGGTTG |
| p37 | AY734921 | 1 | CAACAGCGCGACTTAGTGAA |
|  |  |  | TGGCTTCCACCTTTGTTTTT |
| p46 | AY734930 | 2 | GCGCATTTCTCCATTCATTT |
|  |  |  | GCGGTAATTGTGCGTATGTG |
| p50 | AY734934 | 4 | GTGCAACCAGTGAGCAGTGT |
|  |  |  | TCTGACTGGCCCGTATTTGT |
| p60 | AY734944 | 4 | TACAACCTAGGCAGCCCAAC |
|  |  |  | GTCTGGTTTGGCGATCACTT |
| p66 | AY734950 | 3 | GCAAACCATTTTCCACGAAT |
|  |  |  | CGAAGCATGAATGCAACAAC |
| p70 | AY734954 | 2 | CAGCCTGCCAACACCATT |
|  |  |  | GCAACGCCTTCAAATTCATC |
| p71 | AY734955 | 1 | CGCAAGCACTTTTTGAACTG |
|  |  |  | CTGCTGAATTGGCAGCATAA |
| p73 | AY734957 | 2 | TTTTCTCGTCTACTCGTGTTAGTTAAT |
|  |  |  | AAAATGCACTTTGTAAATAGTCACTCA |
| p80 | AY734964 | 3 | GGACAGTTGTGGTTGCTGAA |
|  |  |  | TCCTTTGCAATGTTATGGTAATTG |
