# Supplementary material for: Hybridization and the spread of the apple maggot fly, Rhagoletis pomonella (Diptera: Tephritidae), in the northwestern United States
Source: Evol Appl. 2015 Aug 13;8(8):834–46. doi: 10.1111/eva.12298 (PMC4561572; doi:10.1111/eva.12298)
Supplement: Supplementary file 6 — Table S4. Mean estimated Ln likelihood, standard deviation, and ΔK (Evanno et al. 2005) calculated across five replicates of STRUCTURE analysis for all eighteen populations for K = 1–5, using a burn-in of 500 000 followed by 1 000 000 MCMC repetitions under a correlated allele frequencies with admixture model. [file eva0008-0834-sd6.docx]

**Supporting Information Table S4**. Mean estimated Ln likelihood, standard deviation, and ΔK (Evanno et al. 2005) calculated across five replicates of STRUCTURE analysis for all eighteen populations for K=1-5, using a burn-in of 500,000 followed by 1,000,000 MCMC repetitions under a correlated allele frequencies with admixture model.

| *K* | *Ln Lik* | *σ* | Δ*K* |
| --- | --- | --- | --- |
| 1 | -44971.9 | 0.13 | N/A |
| 2 | -39169.1 | 0.86 | 6250.02 |
| 3 | -38753.8 | 17.09 | 0.34 |
| 4 | -38332.5 | 3.93 | 36.97 |
| 5 | -38056.6 | 173.04 | 1.59 |
