# Supplementary material for: Hybridization and the spread of the apple maggot fly, Rhagoletis pomonella (Diptera: Tephritidae), in the northwestern United States
Source: Evol Appl. 2015 Aug 13;8(8):834–46. doi: 10.1111/eva.12298 (PMC4561572; doi:10.1111/eva.12298)
Supplement: Supplementary file 7 — Table S5. Mean estimated Ln likelihood and standard deviation across five replicates of a STRUCTURE analysis of paired local R. pomonella and R. zephyria populations for K = 1 and K = 2, using a burn-in of 500 000 followed by 1 000 000 MCMC repetitions under a correlated allele frequency with admixture model. [file eva0008-0834-sd7.docx]

**Supporting Information Table S5.** Mean estimated Ln likelihood and standard deviation across five replicates of a STRUCTURE analysis of paired local *R. pomonella* and *R. zephyria* populations for K=1 and K=2, using a burn-in of 500,000 followed by 1,000,000 MCMC repetitions under a correlated allele frequency with admixture model. “Δ *Ln Lik”* reports the change in mean Ln likelihood between K=2 and K=1. Sites refer to designations in Table S1 and Figure S1.

|  | **K=1** | | **K=2** | |  |
| --- | --- | --- | --- | --- | --- |
| Site | *Ln Lik* | *σ* | *Ln Lik* | *σ* | Δ *Ln Lik* |
| 1) Bellingham | -2320.88 | 1.03 | -2097.6 | 0.16 | -223.28 |
| 2) WSU | -5652.40 | 1.37 | -4803.34 | 0.21 | -849.06 |
| 3) Devine | -9413.26 | 0.59 | -8004.84 | 0.24 | -1408.42 |
| 4) St. Cloud | -6996.36 | 0.78 | -6013.10 | 0.36 | -983.26 |
| 5) Beacon Rock | -5200.92 | 0.40 | -4648.58 | 0.50 | -552.34 |
| 6) Home Valley | -4525.84 | 0.43 | -4067.14 | 0.40 | -458.70 |
| 7) Klickitat | -3355.74 | 0.83 | -3011.06 | 0.63 | -344.68 |
| 8) Burbank/Walla Walla | -1553.70 | 0.25 | -1437.18 | 7.11 | -116.52 |
| 9) Yakima | -2773.58 | 0.49 | -2640.82 | 2.75 | -132.76 |
