# Supplementary material for: Hybridization and the spread of the apple maggot fly, Rhagoletis pomonella (Diptera: Tephritidae), in the northwestern United States
Source: Evol Appl. 2015 Aug 13;8(8):834–46. doi: 10.1111/eva.12298 (PMC4561572; doi:10.1111/eva.12298)
Supplement: Supplementary file 8 — Table S6. Mean demic inbreeding coefficient (f) and stand deviation (σ) across all 19 loci for each of 9 paired R. pomonella and R. zephyria population. [file eva0008-0834-sd8.docx]

**Supporting Information Table S6**. Mean demic inbreeding coefficient (*f*) and stand deviation (σ) across all 19 loci for each of 9 paired *R. pomonella* and *R. zephyria* population. Numbers in parentheses refer to designations in Table S1 and map in Figure S1.

| Location | Species | *f* | σ |
| --- | --- | --- | --- |
| Bellingham (1) | *R. pomonella* | 0.110 | 0.215 |
| Bellingham (1) | *R. zephyria* | 0.192 | 0.227 |
| WSU (2) | *R. pomonella* | 0.160 | 0.190 |
| WSU (2) | *R. zephyria* | 0.089 | 0.256 |
| Devine (3) | *R. pomonella* | 0.206 | 0.167 |
| Devine (3) | *R. zephyria* | 0.098 | 0.201 |
| St.Cloud (4) | *R. pomonella* | 0.096 | 0.217 |
| St. Cloud (4) | *R. zephyria* | 0.164 | 0.274 |
| Beacon Rock (5) | *R. pomonella* | 0.170 | 0.257 |
| Beacon Rock (5) | *R. zephyria* | 0.110 | 0.263 |
| Home Valley (6) | *R. pomonella* | 0.175 | 0.243 |
| Home Valley (6) | *R. zephyria* | 0.155 | 0.257 |
| Klickitat (7) | *R. pomonella* | 0.087 | 0.202 |
| Klickitat (7) | *R. zephyria* | 0.115 | 0.228 |
| Burbank (8) | *R. pomonella* | 0.045 | 0.169 |
| Walla Walla (8) | *R. zephyria* | 0.199 | 0.338 |
| Yakima (9) | *R. pomonella* | 0.113 | 0.265 |
| Yakima (9) | *R. zephyria* | 0.123 | 0.226 |
