# Supplementary material for: Hybridization and the spread of the apple maggot fly, Rhagoletis pomonella (Diptera: Tephritidae), in the northwestern United States
Source: Evol Appl. 2015 Aug 13;8(8):834–46. doi: 10.1111/eva.12298 (PMC4561572; doi:10.1111/eva.12298)
Supplement: Supplementary file 9 — Table S6. Mean demic inbreeding coefficient (f) and stand deviation (σ) across all 19 loci for each of 9 paired R. pomonella and R. zephyria population. [file eva0008-0834-sd9.docx]

**Fig. S1.** STRUCTURE bar plot for analysis of all 18 populations for K=2, the most well-supported number of diverged, non-randomly mating demes based on 19 microsatellites. Analysis was conducted without population priors using the admixture and correlated allele frequency model. Bars along the x-axis represent individual flies and the y-axis represents inferred ancestry in clusters 1 (black) and 2 (light grey). Geographic paired populations are indicated above the plot corresponding to designations in Table S1. P and Z below the plot denote *R. pomonella* and *R. zephyria* flies, respectively.

**Fig. S2**. STRUCTURE bar plots for nine paired sites at (A) Bellingham; (B) WSU; (C) Devine; (D) St. Cloud Park; (E) Beacon Rock State Park; (F) Home Valley; (G) Klickitat; (H) Burbank / Walla Walla, WA; (I) Yakima; depicting posterior probabilities of individual *R. pomonella* black hawthorn fly genotypes (on left) and *R. zephyria* snowberry fly genotypes (on right) belonging to one of four genotypic classes: pure *R. pomonella* origin (black), pure *R. zephyria* (light grey), F1 hybrid (red), or backcross (blue), based on genotypes at 19 microsatellite loci. Bars along the x-axis represent individual flies.
